# Prevalence of genital and extragenital sexually transmitted infections among women of reproductive age with and without HIV in the Southern US: results from the study of treatment and reproductive outcomes

**DOI:** 10.3389/fmed.2025.1537427

**Published:** 2025-03-26

**Authors:** Nicholas F. Nogueira, Laura S. Beauchamps, Yue Pan, Paola Beato Fernandez, Maria Gabriela Rodriguez, Gray Kelsey, Patricia Raccamarich, Candice A. Sternberg, Daniel Westreich, Seble G. Kassaye, Elizabeth F. Topper, Aadia Rana, Deborah Konkle-Parker, Deborah L. Jones, Anandi N. Sheth, Maria L. Alcaide

**Affiliations:** ^1^Division of Infectious Diseases, Department of Medicine, University of Miami Miller School of Medicine, Miami, FL, United States; ^2^Division of Biostatistics, Department of Public Health Sciences, University of Miami Miller School of Medicine, Miami, FL, United States; ^3^Department of Epidemiology, Gillings School of Global Public Health, University of North Carolina at Chapel Hill, Chapel Hill, NC, United States; ^4^Department of Medicine, Georgetown University, Washington, DC, United States; ^5^Department of Epidemiology, Johns Hopkins Bloomberg School of Public Health, Baltimore, MD, United States; ^6^Division of Infectious Diseases, University of Alabama at Birmingham School of Medicine, Birmingham, AL, United States; ^7^Schools of Nursing, Medicine, and Population Health, University of Mississippi Medical Center, Jackson, MS, United States; ^8^Department of Psychiatry and Behavioral Sciences, University of Miami Miller School of Medicine, Miami, FL, United States; ^9^Division of Infectious Diseases, Department of Medicine, Emory University School of Medicine, Atlanta, GA, United States

**Keywords:** sexually transmitted infections, reproductive health, chlamydia, gonorrhea, trichomoniasis, extragenital STIs

## Abstract

**Introduction:**

Sexually transmitted infections (STI) are highly prevalent among women of reproductive age (WRA) and increase the risk of HIV acquisition and transmission. However, the burden of extragenital STIs is understudied among WRA in the US. Estimates of disease are urgently needed among women living with (WWH) and without HIV (WWOH), to inform sex-specific screening guidelines.

**Methods:**

Cross-sectional data from cisgender WWH and WWOH, ages 18–45 years, enrolled in the Study of Treatment and Reproductive Outcomes (STAR) from March 2021 to August 2023 at six Southern US sites was analyzed. Sociodemographic and behavioral assessments were performed using structured interviewer-administered questionnaires. Nucleic-acid amplification tests were performed, regardless of symptoms, on self-collected urine, rectal, and pharyngeal swabs to detect trichomoniasis, chlamydia, and gonorrhea. Sociodemographic characteristics and risk factors were compared by STI status and concordance between genital and extragenital STIs was examined.

**Results:**

Among the 543 participants, 55.2% WWH, mean age was 34.0 (SD ± 7.14) years old, most (72.5%) were non-Hispanic Black, 41.6% had multiple sexual partners, and 85.6% engaged in unprotected sex. Overall, 1.9% tested positive for genital chlamydia, 2.9% rectal chlamydia, 0.6% oropharyngeal chlamydia, 3.4% genital gonorrhea, 1.2% rectal gonorrhea, 1.3% oropharyngeal gonorrhea, and 12.2% trichomoniasis. Genital chlamydia was associated with rectal chlamydia (*p* < 0.001) but not oropharyngeal chlamydia; and genital gonorrhea associated with rectal (*p* < 0.001) and oropharyngeal (*p* = 0.0011) gonorrhea. Eight (11.1%) pregnant participants were diagnosed with at least one STI. Higher genital chlamydia risk was associated with women without healthcare provider visits in the past year [RR = 7.14, 95% CI (1.92, 25.00); *p* = 0.043]; while higher trichomoniasis risk was associated with lower educational attainment of high school or below [RR = 2.94, 95% CI (1.49, 5.88); *p* = 0.009] and an average monthly income of less than $1,500 USD [RR = 4.76, 95% CI (1.82, 12.5); *p* = 0.011]. HIV-status was not associated with genital or extragenital STIs.

**Discussion:**

Prevalence of genital, rectal, oral chlamydia (1.8%, 2.8%, and 0.6%) and gonorrhea (3.3%, 1.1%, and 1.3%), and genital trichomoniasis (11.4%) are high among WRA with and without HIV. The adverse impact for women’s reproductive health and HIV transmission highlights the importance of extragenital STI testing for women in areas of high prevalence of STIs in the US.

## Introduction

Sexually transmitted infections (STIs) are a significant public health concern primarily affecting ethnic and racial minorities and young adults aged 15–24 in the United States (US) (1, 2). Moreover, despite COVID-19 pandemic disruptions in screening in 2020, overall STI reporting increased by 7% from 2017 to 2021 (3–5). Overall, rates of chlamydia decreased by 6.2% (524.6 to 492.2 per 100,000), and gonorrhea increased by 5.2% (170.6 to 179.5 per 100,000) from 2017 to 2023 (6, 7). STIs disproportionately impact young women, contributing to adverse reproductive health and pregnancy outcomes and increased risk of HIV acquisition and transmission (3, 6–12). Among women in the US, rates of chlamydia and gonorrhea have decreased by 1.7 and 14%, respectively, from 2022 to 2023 (6, 7). In contrast to chlamydia and gonorrhea, which are both reportable diseases by the Centers for Disease Control and Prevention (CDC), less data is available for trichomoniasis, a non-notifiable, sexually transmitted protozoal infection commonly referred as the neglected STI, highly prevalent among women with and without HIV In addition (9, 13). Disparities in STIs diagnoses persist globally, with studies suggesting that among women, factors such as age, race, intimate partner violence, childhood abuse, mental health, age of sexual debut, multiple sexual partners, and alcohol are associated with STI diagnoses (14–16).

Women are often underrepresented in clinical research and information on STI burden among reproductive age women remains scarce. This is especially true when considering the context of HIV acquisition and intersectional characteristics between sex and race (17, 18). Among women in the US in 2019, women aged 25–34 and 35–44 had the highest number of new HIV diagnoses, with 54% of new cases occurring in women identifying as Black/African American (19). Women’s risk for HIV can stem from STIs and a variety of other behavioral, biological, and host-related factors ranging from condom use, douching, host mucosal microenvironment, exogenous/endogenous hormones, genital microbiome composition, genital inflammation, amongst others (20). STIs, in particular, exacerbate HIV risk through an elevated pro-inflammatory cytokine response that can cause disruptions to the epithelial barrier (20). In addition to adverse infection outcomes such as pelvic inflammatory disease, infertility, ectopic pregnancy, and preterm delivery, STIs have the potential for vertical transmission during pregnancy and concomitant gonorrhea and chlamydia as well as chlamydia alone has been associated with increased risk of HIV mother-to-child transmission (21–23).

In the Southern United States, the burden of STIs and HIV is particularly high due to a complex interplay of social, economic, and behavioral factors. In 2023, the South had the highest rates of chlamydia (545.3 cases per 100,000; 1.1% decrease from 2022) and gonorrhea (164.3 cases per 100,000; 8.1% decrease from 2022) compared to the Midwest, West, and Northeast regions of the United States (6, 7). Moreover, the South accounted for 52% of new HIV diagnoses in 2021 (19). The prevention of STIs and subsequent avoidance of complications is an area of study that could lead to high impact initiatives in the areas of women’s reproductive health and HIV prevention.

Most clinical discussion emphasizes the diagnosis of genital STIs, with current CDC guidelines recommending annual testing of urogenital chlamydia and gonorrhea in sexually active women younger than 25 years of age and in older women when considering specific risk factors. Screening for trichomonas for people living without HIV is only considered for women seeking care due to symptoms, in high-prevalence settings, or for women engaging in high-risk behaviors (23). Furthermore, despite guidelines recommending regular testing in persons living with HIV, many studies have reported that STI screening remains low, demonstrating potential missed opportunities for treatment (24, 25). Extragenital STIs have been extensively documented among men who have sex with men (MSM), and it has been demonstrated that rectal gonorrhea and chlamydia infections independently increase the risk of HIV acquisition (26). Yet, there is a paucity of research about the prevalence and transmission of rectal gonorrhea and chlamydia among women, and routine testing for extragenital infections is not recommended. In a study examining rectal infections in women reporting ano-receptive intercourse, 17.5% of participants tested positive for rectal chlamydia and 13.4% for rectal gonorrhea (27). However, several studies have found that rectal infections are similarly prevalent among women who do not report receptive anal intercourse, suggesting a need for including routine rectal screening as part of STI and HIV prevention in women (27, 28). Although rarely symptomatic and having low morbidity, far less is known about the prevalence of oropharyngeal STIs in women, which carry implications for disease burden due to transmission from one infection site to another and the development of antimicrobial resistance in the oropharynx (29–31).

Despite significant progress in understanding STI epidemiology and the impact on women’s reproductive health, there remain crucial gaps in knowledge of the underlying mechanisms of genital and extragenital STI disparities for women in the South. Greater understanding of social, behavioral, and structural factors that contribute to the disproportionate burden of STIs among women in the South is needed to develop targeted interventions. This study aims to describe the prevalence of genital chlamydia, gonorrhea, and trichomoniasis, as well as extragenital gonorrhea and chlamydia in the South among reproductive age women living with HIV (WWH) or without HIV (WWOH) enrolled in the Study of Treatment And Reproductive outcomes (STAR), the largest cohort of reproductive age women living with HIV and at risk for HIV in the US.

## Materials and methods

This study is a cross-sectional analysis of baseline data from STAR, a longitudinal observational cohort of 18–45 year old, cisgender WWH, and WWOH (at a ratio of 2 WWH:1 WWOH) who reported one of the following high-risk behaviors within 5 years before enrollment: (1) sex with ≥ 3 men; (2) use of crack, cocaine, opiates, methamphetamines, or injection drug use; (3) STI diagnosis; (4) sex for drugs, money, or shelter; (5) sex with a man living with HIV; or (6) a male sexual partner who reported one of these same risk factors within the 5 years prior to her enrollment, or who had prior incarceration. STAR study sites are Atlanta, GA; Birmingham, AL; Jackson, MS; Chapel Hill, NC; Miami, FL; and Washington, DC.

Detailed methods for participant recruitment, screening, visits, and other study procedures have previously been described (32). A total of 759 women who underwent baseline visits from March 2021 to August 2023 were considered, and 543 individuals who underwent at least one NAAT were included in the analyses. Structured interviews administered during visits consist of self-reported medical, reproductive, and psychosocial history, sexual behavior, COVID-19 experiences, mental health assessments, substance use, and limited physical assessments. Participants undergo nucleic-acid amplification testing (NAAT), regardless of symptoms, on self-collected urine, rectal, and pharyngeal swabs to detect chlamydia, gonorrhea, and trichomoniasis.

Sociodemographic characteristics, sexual behaviors, medical history, and incidence of STIs were tabulated. Chi-square tests of independence and Fisher’s exact tests were used to examine concordance between genital and extragenital STIs. Oropharyngeal and rectal chlamydia were combined as extragenital chlamydia, and oropharyngeal and rectal gonorrhea were combined as extragenital gonorrhea. Factors associated with (1) genital chlamydia, (2) genital gonorrhea, (3) trichomoniasis, (4) extragenital chlamydia, and (5) extragenital gonorrhea were assessed using relative risk estimation by Poisson regression with robust error variance. The following factors were included in the regression analysis: age, race, ethnicity, income, educational attainment, average monthly income, location, doctor visit in past year, HIV status, age at first sexual encounter, number of sexual partners, unprotected sex in the past year, 5-year history of transactional sex, and lifetime gonorrhea, chlamydia, or trichomoniasis diagnosis. Unadjusted relative risk ratios were estimated with 95% confidence intervals (CI) (33). We also estimated ordinary least squares models to assess multicollinearity. Observations with missing values for the response or explanatory variables were excluded. Statistical significance was determined using α = 0.05 and significant results were adjusted with False Discovery Rate (FDR)-corrections for multiple comparisons (34). All analyses were conducted using SAS 9.4 and R version 4.3.2.

## Results

### Sociodemographic characteristics, sexual and STI history, and HIV status

Among the 543 women who underwent at least one NAAT testing, 469 (83.4%) had NAAT results for genital chlamydia, rectal chlamydia, oral chlamydia, genital gonorrhea, rectal gonorrhea, oral gonorrhea, and trichomoniasis, and 74 missed one or more NAAT assessments due to lack of specimen collection, participant refusal, delayed collection until next study visit, or administrative error (Supplementary Tables 1, 2). Participants had a mean age of 34.0 (SD ± 7.14) years old, most, 392 (72.5%), identified as non-Hispanic Black, followed by 66 (12.2%) Hispanic, 52 (9.6%) non-Hispanic White (Table 1). Approximately half, 296 (54.6%), had an educational attainment of high school or lower, 206 (40.5%) earned an average monthly income of less than $1,500 USD, 369 (68.1%) were not married. A large proportion of participants, 482 (89.6%), had seen a health provider in the past year and half, 300 (55.2%) were WWH. Most WWH reported actively taking ART, with 80.9% with a viral load < 200 copies/mL, and median CD4+ T-cell count of 671 (IQR 448–930). Mean age at first sexual encounter was 15.2 (SD ± 3.48), 222 (41.6%) had more than 1 male sexual partner in the past year, and 410 (85.6%) engaged in condomless vaginal or anal sex in the past year. About 10.0% (*n* = 54) had engaged in transactional sex in the past 5 years. Lifetime history of genital chlamydia, gonorrhea, and trichomoniasis were reported by 189 (35.2%), 140 (26.0%), 166 (30.7%) participants respectively. Of the 243 WWOH, only 24 (9.9%) reported a history of lifetime PrEP use, with only 13 (5.4%) reporting PrEP use within the past year.

**TABLE 1:** Sociodemographic, sexual history, and history of STI among study participants by HIV status (*N* = 543).^a^

	HIV status		
**Variable**	**WWOH**	**WWH**	**Total**
	**(*N* = 243)**	**(*N* = 300)**	**(*N* = 543)**
**Age at enrollment—mean (SD)^b^**	32.1 (7.47)	35.5 (6.49)	34.0 (7.14)
**Race and ethnicity—*n* (%)**			
Hispanic	32 (13.2)	34 (11.4)	66 (12.2)
Non-Hispanic Black	160 (66.1)	232 (77.6)	392 (72.5)
Non-Hispanic Other	18 (7.4)	13 (4.3)	31 (5.7)
Non-Hispanic White	32 (13.2)	20 (6.7)	52 (9.6)
**Educational attainment—*n* (%)**			
High school or less	114 (46.9)	182 (60.9)	296 (54.6)
More than high school	129 (53.1)	117 (39.1)	246 (45.4)
**Average monthly income, USD—*n* (%)**			
1,501–3,000	70 (30.6)	87 (31.1)	157 (30.8)
Less than 1,500	79 (34.5)	127 (45.4)	206 (40.5)
More than 3,001	80 (34.9)	66 (23.6)	146 (28.7)
**Marital status—*n* (%)**			
Married	28 (11.5)	54 (18.1)	82 (15.1)
Not married	186 (76.5)	183 (61.2)	369 (68.1)
Other	3 (1.2)	8 (2.7)	11 (2.0)
Widowed, divorced, or separate	26 (10.7)	54 (18.1)	80 (14.8)
**Location—*n* (%)**			
Washington, District of Columbia	37 (15.2)	23 (7.7)	60 (11.0)
Chapel Hill, North Carolina	24 (9.9)	44 (14.7)	68 (12.5)
Atlanta, Georgia	58 (23.9)	75 (25.0)	133 (24.5)
Miami, Florida	62 (25.5)	88 (29.3)	150 (27.6)
Birmingham, Alabama	42 (17.3)	35 (11.7)	77 (14.2)
Jackson, Mississippi	20 (8.2)	35 (11.7)	55 (10.1)
**Pregnant (%)**	20 (8.2)	52 (17.4)	72 (13.3)
**Doctor visit in the past year—*n* (%)**	199 (82.6)	283 (95.3)	482 (89.6)
**Lifetime PrEP use—*n* (%)**	24 (9.9)	NA	24 (4.4)
**PrEP use in the past year—*n* (%)**	13 (5.4)	NA	13 (2.4)
**Age at first sexual encounter—mean (SD)^b^**	15.6 (3.68)	14.9 (3.28)	15.2 (3.48)
**Number of male sexual partners in the past year—*n* (%)**			
0 or 1 male partner	117 (49.2)	195 (65.9)	312 (58.4)
More than 1 male partner	121 (50.8)	101 (34.1)	222 (41.6)
**Unprotected sex in the past year—*n* (%)**	203 (91.4)	207 (80.5)	410 (85.6)
**History of transactional sex in the past 5 years^a^—*n* (%)**	26 (10.7)	28 (9.4)	54 (10.0)
**Current art use—*n* (%)**	NA	294 (98)	NA
**Absolute CD4+ T cells count—median (IQR)^c^**	NA	671 (448–930)	NA
**HIV viral load, < 200 copies/mL—*n* (%)^c^**	NA	157 (80.9)	NA
**Lifetime history of chlamydia—*n* (%)**	90 (37.3)	99 (33.4)	189 (35.2)
**Lifetime history of gonorrhea—*n* (%)**	55 (22.9)	85 (28.5)	140 (26.0)
**Lifetime history of trichomoniasis—*n* (%)**	73 (30.0)	93 (31.2)	166 (30.7)

^a^Individuals with at least one NAAT result were included.

^b^SD, standard deviation.

^c^Missing 4 observations for absolute CD4+ T cells count, and 106 observations for HIV viral load.

### Genital and extragenital STI rates and concordance of STI test results by anatomical site

Ninety-five (17.5%) women were diagnosed with an STI, 82 (15.1%) with genital chlamydia, gonorrhea, or trichomoniasis, and 25 (4.6%) with extragenital chlamydia or gonorrhea. A total of 10 (1.88%) tested positive for genital chlamydia, 15 (2.9%) for rectal chlamydia, and 3 (0.6%) for oropharyngeal chlamydia. Furthermore, 18 (3.38%) tested positive for genital gonorrhea, 6 (1.2%) for rectal gonorrhea, and 7 (1.3%) for oropharyngeal gonorrhea. Lastly, 62 (12.2%) tested positive for trichomoniasis (Table 2). Women who tested positive for genital chlamydia were more likely to test positive for rectal chlamydia (*p* < 0.001) but not oropharyngeal chlamydia (Table 3). Women who tested positive for genital gonorrhea were more likely to test positive for rectal (*p* < 0.001) and oropharyngeal (*p* = 0.0011) gonorrhea (Table 3).

**TABLE 2:** STI results by HIV status (*N* = 543).^a^

	HIV status		
**Variable**	**WWOH**	**WWH**	**Total**
	**(*N* = 243)**	**(*N* = 300)**	**(*N* = 543)**
**Genital chlamydia (*n*—%)**			
Negative	234 (96.3)	289 (96.3)	523 (96.3)
Positive	5 (2.1)	5 (1.7)	10 (1.8)
Missing	4 (1.7)	6 (2.0)	10 (1.8)
**Rectal chlamydia (*n*—%)**			
Negative	225 (92.6)	277 (92.3)	502 (92.5)
Positive	10 (4.1)	5 (1.7)	15 (2.8)
Missing	8 (3.3)	18 (6.0)	26 (4.8)
**Oropharyngeal chlamydia (*n*—%)**			
Negative	237 (97.5)	287 (95.7)	524 (96.5)
Positive	1 (0.4)	2 (0.7)	3 (0.6)
Missing	5 (2.1)	11 (3.7)	16 (3.0)
**Genital gonorrhea (*n*—%)**			
Negative	233 (95.9)	282 (94.0)	515 (94.8)
Positive	5 (2.1)	13 (4.3)	18 (3.3)
Missing	5 (2.1)	5 (1.7)	10 (1.8)
**Rectal gonorrhea (*n*—%)**			
Negative	231 (95.1)	279 (93.0)	510 (93.9)
Positive	3 (1.2)	3 (1.0)	6 (1.1)
Missing	9 (3.7)	18 (6.0)	27 (5.0)
**Oropharyngeal gonorrhea (*n*—%)**			
Negative	236 (97.1)	285 (95.0)	521 (96.0)
Positive	3 (1.2)	4 (1.3)	7 (1.3)
Missing	4 (1.7)	11 (3.7)	15 (2.8)
**Trichomoniasis (*n*—%)**			
Negative	210 (86.4)	235 (78.3)	445 (82.0)
Positive	24 (9.9)	38 (12.7)	62 (11.4)
Missing	9 (3.7)	27 (9.0)	36 (6.6)

^a^Nucleic acid amplification test (NAAT) was performed on self-collected urine, rectal, and oropharyngeal swabs to detect chlamydia. Approximately 74 individuals missed one or more NAAT assessments due to lack of specimen collection, participant refusal, delayed collection until next study visit, or administrative error.

**TABLE 3:** Concordance between genital and extragenital chlamydia and gonorrhea.^a^

Variable	Genital chlamydia		*p*-value
	**Negative**	**Positive**	
Rectal chlamydia (*N* = 508)			**<0.001**^b^
Negative	489 (98.2)	5 (50.0)	
Positive	9 (1.8)	5 (50.0)	
	**Negative**	**Positive**	
Oropharyngeal chlamydia (*N* = 518)			1.000
Negative	506 (99.6)	10 (100.0)	
Positive	2 (0.4)	0 (0)	
**Variable**	**Genital gonorrhea**		***p*-value**
	**Negative**	**Positive**	
Rectal gonorrhea (*N* = 509)			**<0.001**^b^
Negative	490 (99.6)	13 (76.5)	
Positive	2 (0.4)	4 (23.5)	
	**Negative**	**Positive**	
Oropharyngeal gonorrhea (*N* = 521)			**0.0011**^b^
Negative	499 (99.2)	15 (83.3)	
Positive	4 (0.8)	3 (16.7)	

^a^Nucleic Acid Amplification Test (NAAT) was performed on self-collected urine, rectal, and oropharyngeal swabs to detect chlamydia. Only individuals with both results available were included.

^b^Significant *p*-values (<0.05) were bolded.

### STI among pregnant participants

There were 72 pregnant women (Table 4), of whom, eight (11.1%) were diagnosed with at least one STI: two (2.5%) were diagnosed with rectal chlamydia, two (2.5%) with oropharyngeal chlamydia, one (1.3%) with genital gonorrhea, and five (6.3%) with trichomoniasis. More specifically, four had trichomoniasis only, one had rectal chlamydia only, one had genital gonorrhea only, one had both rectal and oral chlamydia, and one had both oral chlamydia and trichomoniasis.

**TABLE 4:** STI results by pregnancy status (*N* = 541).^a^

Variable	Not pregnant	Pregnant	Total
	**(*N* = 469)**	**(*N* = 72)**	**(*N* = 541)**
**Genital chlamydia (*n*—%)**			
Negative	451 (96.2)	70 (13.4)	521 (96.3)
Positive	10 (2.1)	0 (0.0)	10 (1.9)
Missing	8 (1.7)	2 (2.8)	10 (1.9)
**Rectal chlamydia (*n*—%)**			
Negative	431 (91.9)	69 (95.8)	500 (92.4)
Positive	13 (2.8)	2 (2.8)	15 (2.8)
Missing	25 (5.3)	1 (1.4)	26 (4.8)
**Oropharyngeal chlamydia (*n*—%)**			
Negative	452 (96.4)	70 (97.2)	522 (96.5)
Positive	1 (0.2)	2 (2.8)	3 (0.6)
Missing	16 (3.4)	0 (0.0)	16 (3.0)
**Genital gonorrhea (*n*—%)**			
Negative	444 (94.7)	69 (95.8)	513 (94.8)
Positive	17 (3.6)	1 (1.4)	18 (3.3)
Missing	8 (1.7)	2 (2.8)	10 (1.9)
**Rectal gonorrhea (*n*—%)**			
Negative	437 (93.2)	71 (98.6)	508 (93.9)
Positive	6 (1.3)	0 (0.0)	6 (1.1)
Missing	26 (5.5)	1 (1.4)	27 (5.0)
**Oropharyngeal gonorrhea (*n*—%)**			
Negative	447 (95.3)	72 (100.0)	519 (95.9)
Positive	7 (1.5)	0 (0.0)	7 (1.3)
Missing	15 (3.2)	0 (0.0)	15 (2.8)
**Trichomoniasis (*n*—%)**			
Negative	376 (80.2)	67 (93.1)	443 (81.9)
Positive	57 (12.2)	5 (6.9)	62 (11.5)
Missing	36 (7.7)	0 (0.0)	36 (6.7)

^a^Nucleic acid amplification test (NAAT) was performed on self-collected urine, rectal, and oropharyngeal swabs to detect chlamydia. Observations with missing pregnancy status were excluded (*n* = 2).

### Factors associated with genital and extragenital STIs

**FIGURE 1:**
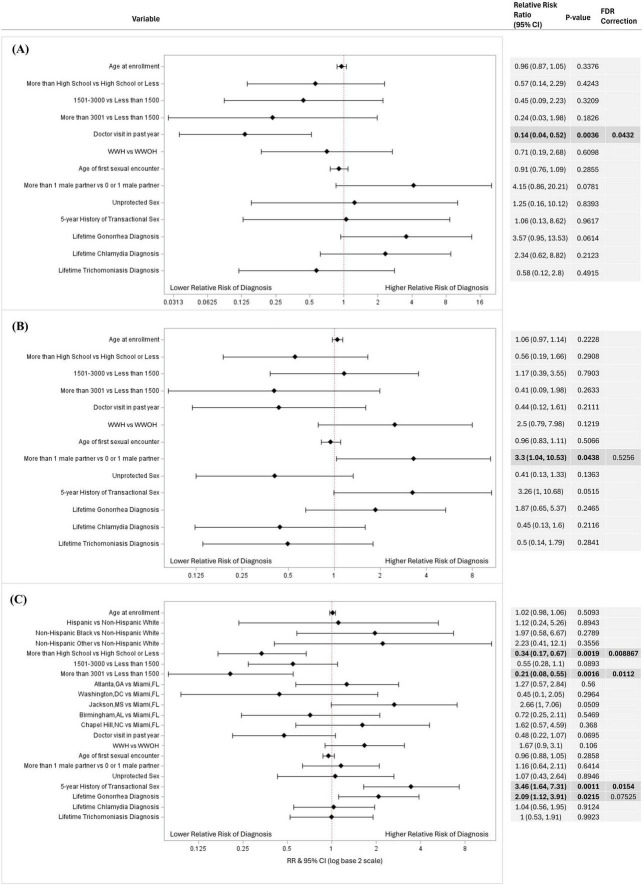
Factors associated with genital **(A)** chlamydia, **(B)** gonorrhea, and **(C)** trichomoniasis diagnosis in univariate logistic regressions. Factors associated with genital STI diagnosis in a univariable logistic regression models for **(A)** chlamydia (*N* = 437), **(B)** gonorrhea (*N* = 437), and **(C)** trichomoniasis (*N* = 416). Variables considered included age at enrollment, race and ethnicity, income, educational attainment, average monthly income, location, doctor visit in past year, HIV status, age at first sexual encounter, number of sexual partners, unprotected sex in the past year, 5-year history of transactional sex, and lifetime gonorrhea, chlamydia, and trichomoniasis diagnosis. Variables that could not be estimated were not included in this figure. Unadjusted relative risk ratios (RRs) were calculated and presented using a log base 2 scale in a Forest Plot. Null line is indicated for no predictor effects and bolded lines represent RRs with 95% confidence intervals. Bolded lines above and below the null line indicate increased or decreased relative risk of STI diagnosis, respectively.

**FIGURE 2:**
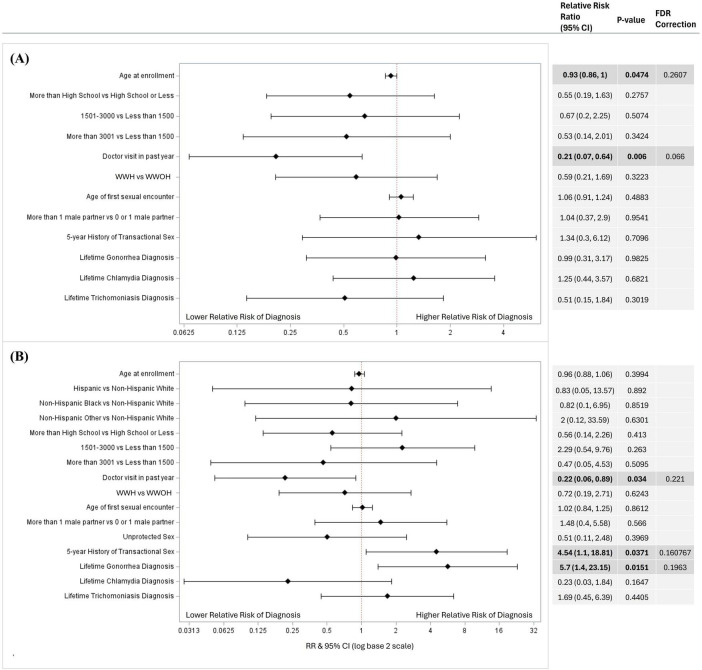
Factors associated with extragenital **(A)** chlamydia and **(B)** gonorrhea diagnosis in univariate logistic regressions. Factors associated with Extragenital STI diagnosis in a univariable logistic regression models for **(A)** chlamydia (*N* = 434), and **(B)** gonorrhea (*N* = 435). Variables considered included age at enrollment, race and ethnicity, income, educational attainment, average monthly income, location, doctor visit in past year, HIV status, age at first sexual encounter, number of sexual partners, unprotected sex in the past year, 5-year history of transactional sex, and lifetime gonorrhea, chlamydia, and trichomoniasis diagnosis. Variables that could not be estimated were not included in this figure. Unadjusted relative risk ratios (RRs) were calculated and presented using a log base 2 scale in a Forest Plot. Null line is indicated for no predictor effects and bolded lines represent RRs with 95% confidence intervals. Bolded lines above and below the null line indicate increased or decreased relative risk of STI diagnosis, respectively.

In relative risk estimation by Poisson regression, a higher risk of genital chlamydia was associated with women who did not visit a healthcare provider in the past year [RR = 7.14, 95% CI (1.92, 25.00); *p* = 0.043] compared to women who did; and a higher risk of trichomoniasis was associated with lower educational attainment of high school or below [RR = 2.94, 95% CI (1.49, 5.88); *p* = 0.009] and an average monthly income of less than $1,500 USD [RR = 4.76, 95% CI (1.82, 12.5); *p* = 0.011] compared to women with an educational attainment of higher than high school and average monthly income of more than $3,001 USD, respectively (Figure 1). After FDR-corrections for multiple comparisons, there were no significant associations between the variables included in the models and oropharyngeal/rectal chlamydia or oropharyngeal/rectal gonorrhea (Figure 2). HIV status was associated with neither genital nor extragenital STIs.

## Discussion

This study evaluated genital and extragenital STIs among WWH and WWOH in six Southern cities in the US, and enrolled in STAR, the largest cohort of reproductive age women with HIV and demographically similar women without HIV in the US. Results indicated relatively high prevalence of genital, rectal, oral chlamydia (1.8, 2.8, and 0.6%) and gonorrhea (3.3, 1.1, and 1.3%), and genital trichomoniasis (11.4%) in women with and without HIV. Education, income, and transactional sex were associated with a higher risk of trichomoniasis, and lack of prior healthcare visits was associated with higher risk of genital chlamydia.

STIs are often asymptomatic and may thus remain undiagnosed and untreated for long periods of time. Lack of treatment can result in severe reproductive health outcomes which are particularly important for younger women. Gonorrhea and chlamydia are associated with pelvic inflammatory disease as well as ectopic pregnancy (35, 36). The consequences of a STI during pregnancy can expand beyond maternal heath to neonatal outcomes, since gonorrhea, chlamydia and trichomoniasis have demonstrated increased risk of preterm birth (9, 10). The higher risk of genital chlamydia among women who did not visit a healthcare provider in the past year highlights the pivotal role clinical providers may play in STI diagnosis, treatment, and prevention. Although STIs have been associated with risk of HIV acquisition, we found no differences in prevalence of genital and extragenital infection by HIV status (37). This could be because WWOH reported engaging with more risky behaviors such as more than 1 male sexual partner and unprotected sex in the past year. Alternatively, this study found that WWOH had lower healthcare utilization, which may indicate less exposure or limited access to STI testing compared to WWH. Novel efforts to expand the reach of providers in STI testing programs to communities with high rates of HIV or vulnerability to HIV remain critical to further lower the incidence of STIs, the consequences in reproductive health, and to limit the spread of HIV.

This study adds to the limited data evaluating extragenital STIs in women in the US. Prior studies have shown high prevalence among women who report anal sex, but this study is among the few in which extragenital chlamydia and gonorrhea testing were performed independent of anal sexual activity and includes pregnant women with and without HIV. Previous studies examining STIs in women have reported high prevalence of rectal chlamydia, 2.0–77.3%, oropharyngeal chlamydia, 0.2–3.2%, rectal gonorrhea, 0.6–35.8%, and oropharyngeal gonorrhea, 0–29.6%, and a discrepancy of genital and extragenital testing (27, 28, 38). Despite concerns of alarming increasing of STI rates, and an increase rate of extragenital STIs, routine extragenital screening is still not recommended for women in current STI guidelines and testing remains suboptimal (39, 40). Although symptomatology was not evaluated in this study, relying solely on symptoms or disclosure of risky sexual behaviors to determine when extragenital testing is conducted may fail to capture cases, especially in high-prevalence settings, as they are often asymptomatic and would lead to undiagnosed and untreated infections and will result in increased risk of STI and HIV transmission, and poor reproductive health outcomes (36). Moreover, incorporating extragenital testing in routine guidelines may also aid in normalizing conversations surrounding STIs and oral/anal concerns between patients and providers, lowering stigma around STI screenings and improving sexual health outcomes. Our study confirms that genital and extragenital STIs are common among women and reinforces the importance of including extragenital sites in routine STI testing for women living with HIV and at risk for HIV.

*Trichomonas vaginalis*, should not remain a neglected STI as its sequelae in women include increased risk of HIV acquisition, cervical cancer, preterm birth, and other adverse pregnancy outcomes. A meta-analysis of 19 peer-reviewed studies found that persons with *T. vaginalis* were 1.5 times more likely to acquire HIV, and WWH have higher incidence of trichomoniasis than WWOH (13, 41). Despite having a higher global prevalence than that of chlamydia, gonorrhea, and syphilis combined, only two population-based studies exist examining trichomoniasis infection occurring in the United States (41, 42). Data obtained in the National Health and Nutrition Examination Survey (NHANES) from 2013 to 2016 demonstrated a 4-fold higher prevalence of trichomoniasis in women compared to men, and an 11-fold higher prevalence in Black non-Hispanic females. Furthermore, CDC estimates U.S. population-based prevalence of Trichomoniasis at 2.1% among women, with higher rates, 9.6%, among black women and larger estimates, 14.6–27%, in STD clinics (43). Notwithstanding, we found that race was not associated with risk of a trichomoniasis diagnosis but rather other social determinants of health (44). Cis-gender female sex workers have previously been found to have an elevated risk of trichomoniasis diagnosis, consistent with higher rates of trichomoniasis among women engaging in transactional sex in the past 5 years (45). This study found that lower levels of education, lower income, and history of transactional sex were associated with a trichomoniasis diagnosis, and as a result interventions targeting these groups to decrease the incidence of trichomoniasis may be warranted, especially when implementing programs to decrease new HIV and improve reproductive health outcomes. Low income and low education areas would benefit from comprehensive STI and HIV testing, including rapid (or point of care) HIV and *T. vaginalis* testing as well as culturally tailored education on STI and HIV prevention (46). Similarly, health disparities play a critical role in the prevalence of chlamydia and gonorrhea, especially in the Southern US. Although age and race were not associated with chlamydia and gonorrhea prevalence in this study, previous studies reported higher prevalence of both among individuals identifying as non-Hispanic Black and among adolescents and young adults aged 15–24, possibly due to differences in sexual health care access and sexual networks (7, 47). For instance, year-round uninsurance has been associated with lower odds of STI testing compared to those with continuous private coverage [OR = 0.37, 95% CI (0.25–0.55)] (48). Considering the intersectionality between health disparities, STIs, and HIV, efforts towards education and testing delivery should be concentrated at local health centers of low education and low-income areas or through mobile care units, to augment diagnosis and access to testing in WRA vulnerable to acquisition of HIV.

This study also highlights further needs for additional preventive strategies for STIs. New digital health interventions (e.g., telehealth, dating app-based interventions) and at-home self-testing STI kits could provide an alternative approach to augment STI education and coordination of testing services, while keeping in mind challenges to implementation and access to these systems (49–54). Leveraging existing infrastructure in healthcare departments and federal/state programs, such as managed care organization with Medicaid or state specific legislation, to expand on STI testing coverage also provides an additional avenue to improve health outcomes (51, 55). Findings of this study also revealed low uptake of pre-exposure prophylaxis (PrEP) further illustrating the need to increase PrEP awareness, uptake, and retention of oral and long-acting injection PrEP among reproductive age women. Thus, the use of multipurpose prevention technologies by simultaneously addressing gaps in PrEP use and STI testing could be beneficial when bundled with multiple services like HIV prevention, treatment, contraception, and substance use programs in STI, OGBYN, family planning, and primary clinics (51, 56–59). In addition, doxycycline post-exposure prophylaxis (DoxyPEP) is a promising strategy for STI reduction, yet early studies reveal no effectiveness in decreasing STIs among women (60–62). Further studies are warranted to identify reasons for lack of efficacy of DoxyPEP and use and optimize STI prevention interventions tailored for cisgender women.

This study has some limitations. Most of the women in the sample identified as non-Hispanic Black in the U.S. South and as a result, the results may not be comparable to other populations. Yet, a strength of the sample is the large representation of Black women, particularly given their risk of poor reproductive outcomes and racial disparities with regard to mortality during childbirth in the United States (63). Due to the limited number of oropharyngeal and rectal STI diagnoses, this study was not able to explore associations with these diagnoses independently of the other, and interviewer-administered questionnaires may be subjected to social desirability bias in self-reported answers surrounding sensitive topics like sexual health and risk behaviors. Furthermore, cross-sectional analyses cannot establish causality, may be subjected to reverse causality, and may underrepresent incidence of STIs as prevalence is a combination of incidence, persistence, and survival (64).

This study has considerable strengths showcasing areas of further focus for STI prevention. Overall, prevalence and incidence of genital STIs remain high, and it extragenital STI in women is unknown according to national statistics. Potential areas of improvement through policy changes is the inclusion of universal extragenital STI testing in national guidelines in high-prevalence settings and among WRA engaging in high risk behaviors, and designating trichomoniasis as a notifiable disease. These may be critical to improving the epidemiological landscape of STIs and would have positive implications in improving reproductive health outcomes and decreased HIV transmission. Future longitudinal studies are warranted to evaluate the impact of health disparities in STI persistence and transmission.

## Data Availability

The raw data supporting the conclusions of this article will be made available by the authors, without undue reservation.
