# Supplementary material for: Prevalence of genital and extragenital sexually transmitted infections among women of reproductive age with and without HIV in the Southern US: results from the study of treatment and reproductive outcomes
Source: Front Med (Lausanne). 2025 Mar 26;12:1537427. doi: 10.3389/fmed.2025.1537427 (PMC11978642; doi:10.3389/fmed.2025.1537427)
Supplement: Supplementary file 1 [file Table_1.docx]

| **Supplementary Table 1.** Sociodemographic, sexual history, and history of STI by STIs among study participants | | | | | | | | | |
| --- | --- | --- | --- | --- | --- | --- | --- | --- | --- |
|  | **Genital Chlamydia**  **(N=533)** | | | **Genital Gonorrhea**  **(N=533)** | | | **Trichomoniasis**  **(N=507)** | | |
| **Variable** | **No** | **Yes** | **p-value** | **No** | **Yes** | **p-value** | **No** | **Yes** | **p-value** |
|  | **(N=523)** | **(N=10)** |  | **(N=515)** | **(N=18)** |  | **(N=445)** | **(N=62)** |  |
| **Age at Enrollment - mean (SD)^1^** | 34.0 (7.09) | 32.8 (9.59) | 0.605 | 33.9 (7.13) | 36.9 (7.29) | 0.08 | 33.6 (7.19) | 35.5 (6.94) | 0.15* |
| **Race & Ethnicity - n (%)** |  |  | 0.688 |  |  | 0.995 |  |  | 0.359 |
| Hispanic | 65 (12.5) | 1 (10.0) |  | 64 (12.5) | 2 (11.1) |  | 61 (13.7) | 5 (8.1) |  |
| Non-Hispanic Black | 375 (72.0) | 8 (80.0) |  | 371 (72.3) | 13 (72.2) |  | 313 (70.5) | 48 (77.4) |  |
| Non-Hispanic White | 29 (5.6) | 1 (10.0) |  | 29 (5.7) | 1 (5.6) |  | 24 (5.4) | 5 (8.1) |  |
| Non-Hispanic Other | 52 (10.0) | 0 (0) |  | 49 (9.6) | 2 (11.1) |  | 46 (10.4) | 4 (6.5) |  |
| **Educational Attainment - n (%)** |  |  | 0.321 |  |  | 0.292 |  |  | **0.008*** |
| High School or Less | 283 (54.2) | 7 (70.0) |  | 278 (54.1) | 12 (66.7) |  | 232 (52.3) | 46 (74.2) |  |
| More than High School | 239 (45.8) | 3 (30.0) |  | 236 (45.9) | 6 (33.3) |  | 212 (47.7) | 16 (25.8) |  |
| **Average Monthly Income, USD - n (%)** |  |  | 0.338 |  |  | 0.208 |  |  | **0.02*** |
| Less than 1500 | 153 (31.3) | 3 (30.0) |  | 148 (30.6) | 8 (47.1) |  | 134 (32.1) | 14 (24.6) |  |
| 1501-3000 | 196 (40.1) | 6 (60.0) |  | 195 (40.4) | 7 (41.2) |  | 156 (37.3) | 34 (59.6) |  |
| More than 3001 | 140 (28.6) | 1 (10.0) |  | 140 (29.0) | 2 (11.8) |  | 128 (30.6) | 9 (15.8) |  |
| **Marital Status - n (%)** |  |  | 0.87 |  |  | 0.073 |  |  | 0.632 |
| Not Married | 78 (14.9) | 1 (10.0) |  | 74 (14.4) | 4 (22.2) |  | 67 (15.1) | 6 (9.7) |  |
| Married | 356 (68.2) | 8 (80.0) |  | 356 (69.3) | 8 (44.4) |  | 299 (67.3) | 45 (72.6) |  |
| Widowed, Divorced, or Separated | 10 (1.9) | 0 (0) |  | 11 (2.1) | 0 (0) |  | 9 (2.0) | 2 (3.2) |  |
| Other | 78 (14.9) | 1 (10.0) |  | 73 (14.2) | 6 (33.3) |  | 69 (15.5) | 9 (14.5) |  |
| **Location - n (%)** |  |  | **0.03*** |  |  | 0.274 |  |  | 0.132 |
| Washington, District of Columbia | 124 (23.7) | 7 (70.0) |  | 125 (24.3) | 6 (33.3) |  | 106 (23.8) | 16 (25.8) |  |
| Chapel Hill, North Carolina | 58 (11.1) | 0 (0) |  | 56 (10.9) | 1 (5.6) |  | 50 (11.2) | 3 (4.8) |  |
| Atlanta, Georgia | 148 (28.3) | 0 (0) |  | 143 (27.8) | 5 (27.8) |  | 132 (29.7) | 17 (27.4) |  |
| Miami, Florida | 52 (9.9) | 0 (0) |  | 49 (9.5) | 4 (22.2) |  | 32 (7.2) | 9 (14.5) |  |
| Birmingham, Alabama | 77 (14.7) | 0 (0) |  | 75 (14.6) | 2 (11.1) |  | 70 (15.7) | 6 (9.7) |  |
| Jackson, Mississippi | 64 (12.2) | 3 (30.0) |  | 67 (13.0) | 0 (0) |  | 55 (12.4) | 11 (17.7) |  |
| **Doctor Visit in the past year - n (%)** | 467 (90.0) | 6 (60.0) | **0.03*** | 459 (89.8) | 15 (83.3) | 0.375 | 396 (89.6) | 50 (83.3) | 0.148 |
| **Persons living with HIV - n (%)** | 289 (55.3) | 5 (50.0) | 0.741 | 282 (54.8) | 13 (72.2) | 0.143 | 235 (52.8) | 38 (61.3) | 0.209 |
| **Age at First Sexual Encounter - mean (SD)^1^** | 15.2 (3.48) | 14.2 (3.52) | 0.355 | 15.2 (3.49) | 14.6 (2.03) | 0.476 | 15.2 (3.50) | 15.0 (3.29) | 0.602 |
| **Number of Male Sexual Partners in the past year - n (%)** |  |  | 0.072 |  |  | 0.094 |  |  | 0.684 |
| 0 or 1 male partner | 300 (58.4) | 3 (30.0) |  | 297 (58.7) | 7 (38.9) |  | 251 (57.6) | 34 (54.8) |  |
| More than 1 male partner | 214 (41.6) | 7 (70.0) |  | 209 (41.3) | 11 (61.1) |  | 185 (42.4) | 28 (45.2) |  |
| **Unprotected Sex in the past year - n (%)** | 394 (85.3) | 8 (88.9) | 0.762 | 390 (85.7) | 11 (73.3) | 0.183 | 338 (85.6) | 46 (85.2) | 0.94 |
| **History of Transactional Sex in the past 5 years^1^ - n (%)** | 53 (10.2) | 1 (10.0) | 0.987 | 50 (9.7) | 4 (22.2) | 0.084 | 38 (8.6) | 14 (22.6) | **<0.001** |
| **Lifetime History of Chlamydia - n (%)** | 182 (35.2) | 5 (50.0) | 0.333 | 184 (36.1) | 4 (22.2) | 0.228 | 152 (34.5) | 22 (36.1) | 0.806 |
| **Lifetime History of Gonorrhea - n (%)** | 134 (25.8) | 5 (50.0) | 0.085 | 134 (26.2) | 6 (33.3) | 0.502 | 105 (23.7) | 24 (40.0) | 0.007 |
| **Lifetime History of Trichomoniasis - n (%)** | 163 (31.3) | 2 (20.0) | 0.445 | 160 (31.2) | 5 (27.8) | 0.759 | 131 (29.6) | 19 (30.6) | 0.862 |
| *Note.*  ^1^SD: Standard deviation.  *FDR adjustments reported instead of the unadjusted, significant p-values. | | | | | | | | | |

| **Supplementary Table 2.** Sample Characteristics & Sexual Behaviors by Extragenital STIs | | | | | | |
| --- | --- | --- | --- | --- | --- | --- |
|  | **Extragenital Chlamydia**  **(N=533)** | | | **Extragenital Gonorrhea**  **(N=533)** | | |
| **Variable** | **No** | **Yes** | **p-value** | **No** | **Yes** | **p-value** |
|  | **(N=516)** | **(N=17)** |  | **(N=523)** | **(N=10)** |  |
| **Age at Enrollment - mean (SD)^1^** | 34.1 (7.08) | 30.4 (8.52) | 0.248* | 34.0 (7.15) | 33.0 (8.10) | 0.659 |
| **Race & Ethnicity - n (%)** |  |  | 0.346 |  |  | 0.264 |
| Hispanic | 62 (12.1) | 3 (17.6) |  | 65 (12.5) | 1 (10.0) |  |
| Non-Hispanic Black | 372 (72.4) | 12 (70.6) |  | 378 (72.6) | 6 (60.0) |  |
| Non-Hispanic White | 28 (5.4) | 2 (11.8) |  | 28 (5.4) | 2 (20.0) |  |
| Non-Hispanic Other | 52 (10.1) | 0 (0) |  | 50 (9.6) | 1 (10.0) |  |
| **Educational Attainment - n (%)** |  |  | 0.166 |  |  | 0.315 |
| High School or Less | 276 (53.6) | 12 (70.6) |  | 282 (54.0) | 7 (70.0) |  |
| More than High School | 239 (46.4) | 5 (29.4) |  | 240 (46.0) | 3 (30.0) |  |
| **Average Monthly Income, USD - n (%)** |  |  | 0.621 |  |  | 0.295 |
| Less than 1500 | 150 (31.1) | 5 (31.3) |  | 150 (30.6) | 5 (50.0) |  |
| 1501-3000 | 193 (40.0) | 8 (50.0) |  | 197 (40.2) | 4 (40.0) |  |
| More than 3001 | 140 (29.0) | 3 (18.8) |  | 143 (29.2) | 1 (10.0) |  |
| **Marital Status - n (%)** |  |  | 0.253 |  |  | 0.325 |
| Not Married | 81 (15.7) | 0 (0) |  | 80 (15.3) | 1 (10.0) |  |
| Married | 347 (67.4) | 13 (76.5) |  | 353 (67.6) | 7 (70.0) |  |
| Widowed, Divorced, or Separated | 11 (2.1) | 0 (0) |  | 10 (1.9) | 1 (10.0) |  |
| Other | 76 (14.8) | 4 (23.5) |  | 79 (15.1) | 1 (10.0) |  |
| **Location - n (%)** |  |  | 0.192 |  |  | 0.767 |
| Washington, District of Columbia | 124 (24.0) | 7 (41.2) |  | 128 (24.5) | 2 (20.0) |  |
| Chapel Hill, North Carolina | 57 (11.0) | 0 (0) |  | 55 (10.5) | 1 (10.0) |  |
| Atlanta, Georgia | 140 (27.1) | 6 (35.3) |  | 145 (27.7) | 3 (30.0) |  |
| Miami, Florida | 54 (10.5) | 1 (5.9) |  | 53 (10.1) | 2 (20.0) |  |
| Birmingham, Alabama | 76 (14.7) | 0 (0) |  | 74 (14.1) | 2 (20.0) |  |
| Jackson, Mississippi | 65 (12.6) | 3 (17.6) |  | 68 (13.0) | 0 (0) |  |
| **Doctor Visit in the past year - n (%)** | 461 (90.2) | 12 (70.6) | 0.135* | 466 (90.0) | 7 (70.0) | 0.205* |
| **Persons living with HIV - n (%)** | 287 (55.6) | 7 (41.2) | 0.239 | 288 (55.1) | 5 (50.0) | 0.75 |
| **Age at First Sexual Encounter - mean (SD)^1^** | 15.2 (3.50) | 15.6 (3.30) | 0.582 | 15.2 (3.48) | 16.0 (2.54) | 0.443 |
| **Number of Male Sexual Partners in the past year - n (%)** |  |  | 0.984 |  |  | 0.586 |
| 0 or 1 male partner | 297 (58.6) | 10 (58.8) |  | 301 (58.6) | 5 (50.0) |  |
| More than 1 male partner | 210 (41.4) | 7 (41.2) |  | 213 (41.4) | 5 (50.0) |  |
| **Unprotected Sex in the past year - n (%)** | 387 (85.4) | 16 (100) | 0.1 | 396 (86.1) | 7 (77.8) | 0.478 |
| **History of Transactional Sex in the past 5 years - n (%)** | 51 (9.9) | 2 (11.8) | 0.801 | 50 (9.6) | 3 (30.0) | 0.248* |
| **Lifetime History of Chlamydia - n (%)** | 179 (35.0) | 7 (41.2) | 0.597 | 184 (35.5) | 1 (10.0) | 0.095 |
| **Lifetime History of Gonorrhea - n (%)** | 135 (26.4) | 5 (29.4) | 0.78 | 133 (25.6) | 7 (70.0) | **0.03*** |
| **Lifetime History of Trichomoniasis - n (%)** | 158 (30.7) | 3 (17.6) | 0.248 | 156 (29.9) | 5 (50.0) | 0.172 |
| *Note.*  ^1^SD: Standard deviation.  *FDR adjustments reported instead of the unadjusted, significant p-values. | | | | | | |
